# The role of pseudotype neutralization assays in understanding SARS CoV-2

**DOI:** 10.1093/oxfimm/iqab005

**Published:** 2021-03-13

**Authors:** Diego Cantoni, Martin Mayora-Neto, Nigel Temperton

**Affiliations:** Viral Pseudotype Unit, Medway School of Pharmacy, The Universities of Kent and Greenwich at Medway, Chatham, ME7 4TB, UK

**Keywords:** SARS-CoV-2 pseudotype, pseudotype neutralization assays

Neutralization assays are crucial tools to quantify the presence of functional neutralizing antibodies in serum samples. Since the SARS-CoV-2 virus (the causative agent of COVID-19) is designated as a Category 3 biosafety level pathogen, pseudotyped viruses (PVs) bearing the SARS-CoV-2 spike protein permit extensive and widespread serum/plasma screening in a BSL 2 laboratory. These assays can be used to assess viral tropism, vaccine immunogenicity, efficacy of antiviral compounds (including therapeutic mAbs) and serosurveillance studies. In this article, we highlight approaches to SARS-CoV-2 viral pseudotyping, its practicality, and utility in increasing our understanding of neutralizing antibodies against SARS-CoV-2.

**Figure:**
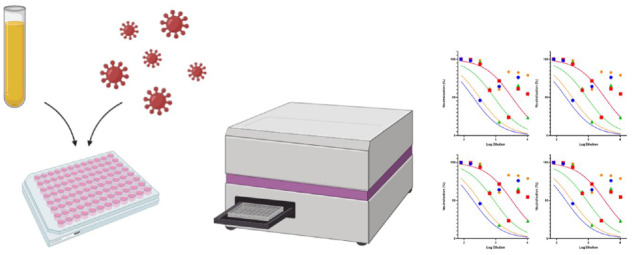


PVs are an alternative to using wild-type viruses for investigating viral entry and tropism. The structural core of one virus is used with the surface glycoprotein(s) of another virus, greatly reducing the risk associated with handling the wild-type virus and thus removing the requirement for high-containment laboratories. Several types of viral cores are used for generating PVs such as murine leukaemia virus, vesicular stomatitis virus and, human immunodeficiency virus (HIV), all of which have been described previously [1–5]. The core of lentiviral PVs contains a retroviral vector with a reporter that, upon transduction of target host cells, is integrated into the cellular genome and expressed. A luciferase or a fluorescent protein (GFP) is typically used as the reporter gene, although colorimetric methods of assay readout exist, which would negate the need for expensive laboratory hardware [6, 7]. It is worth mentioning that due to the incorporation of only the viral surface protein (such as Spike), this system is not able to measure the efficacy of neutralization to other structural viral proteins, since they are not incorporated into the pseudotype viruses.

Typical PV generation and titration can be carried out within a 2-week timeframe and may be readily scaled up to produce large volumes of virus. Storage is recommended at −80°C or −20°C for a short period of time with little loss of titre, and freeze thaws are preferably kept to a minimum, though some PVs may not lose much infectivity even after three freeze-thaw cycles [8, 9]. Various methodologies are publicly available that describe in detail the steps required to generate SARS-CoV-2 PVs [9–12]. The PV neutralization assay (PVNA) is typically carried out in 96-well plates, whereby serum samples are serially diluted and then incubated with a fixed amount of SARS-CoV-2 PVs to allow any neutralizing antibodies to bind to the Spike (S). Cells naturally expressing the ACE2 receptor and the transmembrane serine protease 2 (TMPRSS2) are then added to the plates and incubated for 48–72 h otherwise co-transfection of ACE2 and TMPRSS2 plasmids can be carried out to render cells such as HEK 293T, and HeLa permissive to infection. If the PVs contain a luciferase reporter, then cells are lysed, and luciferase reporter expression is quantified using a luminometer. For GFP-based assays, the GFP positive cells are counted at each dilution to determine the percentage of neutralization, using a fluorescent microscope or a flow cytometer.

The benefits of using a SARS-CoV-2 PV aside from the enhanced biosafety are that PVNA screening is highly serum sparing, with as little as 2µL per sample required, and that the assay itself is based on the spike S protein being the only CoV antigen in the system, therefore validating antibody-receptor binding and neutralization. While it is true that ELISA assays can be used to detect presence of antibodies in response to viral infection, the system itself specifically detects antibody binding to the viral protein, but it does not measure whether this interaction results in neutralization, though recently next-generation kits have been developed to provide that level of information [13–15]. Typically, an initial screening would start with ELISA to detect serum samples with antibodies, followed by a PVNA, which would then provide information on antibody neutralization. PVNAs have been shown to correlate broadly with wild-type virus neutralization assays [2, 12, 16, 17].

Furthermore, generating mutant spike protein can be done readily by site-directed mutagenesis, allowing to produce SARS-CoV-2 PVs bearing mutants that may be in circulation (such as the variants of concern for SARS-CoV-2), or to evaluate the importance of specific amino acids on receptor binding and entry [18–20]. The relative ease and short time frame involved in generating single-point mutations allows for the rapid screening of key sites and highlights the malleability of PVs and their use for investigating variants in circulation [21–23]. It is for these reasons that PVNA are considered to be highly valuable tools in investigating efficacy of antibody responses to viruses.

Many research reports have incorporated PVNA to detect the presence of neutralizing antibodies against SARS-CoV-2. The verification of the first human monoclonal antibody that neutralizes both SARS-CoV-1 and SARS-CoV-2, 47D11, was assessed using PVNAs [24]. This particular study was illuminating as 47D11 did not prevent Spike binding to ACE2, suggesting an unknown mechanism for neutralization. Nevertheless, strong cross-neutralization activity was detected in both PVNAs and wild-type virus assays, even though 47D11 does not compromise SARS-CoV-2 spike-ACE2 receptor binding. This highlights the strengths of using PVNA, as it can detect neutralizing activity of antibodies that do not necessarily prevent virus-host receptor binding. In another study, authors examined plasma samples from patients that had previously travelled from Wuhan to Shenzhen, China, during the outbreak using pseudotyped HIV particles with either SARS-CoV-1, SARS-CoV-2 or MERS-CoV [25]. Some of the patients in this study comprised a family cluster, including the first documented case of human to human transmission in Shenzhen [26]. Neutralizing antibody levels varied between patients, and there was little cross-neutralization between the different PVs, suggesting that the RBDs between SARS-CoV-1 and MERS-CoV are immunologically distinct. The authors had additionally compared the antibody neutralization results using wild-type virus, which was consistent with the PV assay data. A study conducted early during the outbreak at a children’s hospital in Seattle, WA, USA had used PV to screen for neutralizing antibodies [27]. Overall, 10 seropositive samples had SARS-CoV-2 neutralizing antibodies, and seven of the seropositive samples were collected from six children who had never tested positive for SARS-CoV-2. We found this to be a strong example of how effective PVNAs can be as a serological screening tool to detect individuals carrying neutralizing antibodies to SARS-CoV-2 despite testing negative by PCR. This approach of using PVs as a screening tool can be scaled up as part of larger serological surveillance studies [28].

PVNA can also be used to measure neutralizing antibody responses in patients with varying severity of disease [29, 30]. Sera from recovered COVID-19 patients from a hospital in Guangzhou, China categorized as severe, moderate and mild, were subjected to PVNAs [31]. Within the study, asymptomatic sera from patients from Chongqing, China, were also included. The results showed a correlation between serum neutralization capacity and disease severity, whereby high severity had most neutralizing activity. The asymptomatic samples, however, showed no ability to neutralize PV activity, despite detection of RBD-binding antibodies by ELISA. The pseudotyped nAb assay results were then confirmed with wild-type virus experiments. This highlights the strengths of using PVs as a surrogate virus for neutralization assays. Studies have also incorporated PVNAs as part of longitudinal studies on antibody responses. One study in the UK investigated antibody responses in COVID-19 positive patients that were categorized by a severity score ranging from 0; asymptomatic, to 5; requirement for extracorporeal membrane oxygenation [32]. The pseudotyped nAb assays were able to reveal that disease severity enhanced the magnitude of neutralizing antibody response and the authors were able to monitor the neutralization response in samples collected over 50 days post onset of symptoms. Similar longitudinal studies also reported comparable findings using either PVNAs or wild-type virus, which provides insight into the kinetics of the immune response, starting at onset of infection with follow up bleeds over time [33, 34].

Due to the urgent need for a suite of effective vaccines to protect individuals globally against SARS-CoV-2, significant developments are being made, and many published reports have also incorporated PVNA as part of the toolbox to investigate antibody responses post immunization in both human and animal models [35, 36]. For example, in the Phase 1 trial of the mRNA-1273 candidate vaccine, PVNAs were employed before and after each round of vaccination [37]. PVNAs revealed modest antibody responses after the first round of vaccination, followed by a robust antibody response after the second round of vaccination. Similarly, another study investigating humoral immune responses in mice after initial immunization using nucleoside-modified mRNA vaccines observed efficient PV neutralization from the antibodies in the sera [38]. Another investigation developed a prototype DNA vaccine, which was tested in rhesus macaques. The PVNAs revealed strong inhibition by the antibodies in the sera, similar to the levels observed in patients that recover from SARS-CoV-2, which was verified by ELISA and wild-type virus [39]. An investigation to assess effectiveness of the immune response after a second infection in rhesus macaques had used both PVNAs and wild-type viral neutralization assays, of which both revealed that the second challenge resulted in significant decrease of viral load compared to the initial viral infection [40].

Ongoing studies are aiming to use PVNAs to address a key question; what are the immune correlates of protection? Currently, the World Health Organization and National Institute for Biological Standards and Control (NIBSC) have developed a serological standard in order to allow studies to report antibody responses in international units, which would provide valuable insight into what constitutes a protective immune response in individuals [41]. In conclusion, we believe that PVs are a simple and valuable tool to investigate the presence of neutralizing antibodies in serum samples from animal models and humans. These assays can be scaled to allow high-throughput screening and are safe to carry out in BSL-2 laboratories.

## DATA AVAILABILITY

There is no data associated with this review article.

## FUNDING

The Temperton group is funded by the Wellcome Trust (GB-CHC-210183), the MRC (MC_PC_19060) and MRC/NIHR (MC_PC_20016).
